# The Relationship Between Reactive Agility and Change of Direction Speed in Professional Female Basketball and Handball Players

**DOI:** 10.3389/fpsyg.2021.708771

**Published:** 2021-09-30

**Authors:** Marek Popowczak, Ireneusz Cichy, Andrzej Rokita, Jarosław Domaradzki

**Affiliations:** Faculty of Physical Education and Sport, Wroclaw University of Health and Sport Sciences, Wroclaw, Poland

**Keywords:** competitive sport, cognitive motor skills, team sport, performance analysis, motor performance

## Abstract

Assessing the physical ability of players to perform change of direction and the cognitive and motor abilities revealed in reactive agility (RA) is necessary to understand the physical requirements and capabilities of professional players in handball and basketball. The main aim of this study was to determine the differences between professional female basketball and handball players in terms of anthropometric features, change of direction speed (CODS), and the RA task. Moreover, the relationships among anthropometric features, agility, and parameters of perception were determined. Two scenarios of the Five-Time Shuttle Run to Gates test (planned and unplanned) were used to evaluate the CODS and RA. The response time (RT) was also measured in the unplanned scenario. Additionally, the index of reactivity (REAC-INDEX) was specified as the difference between the RA test result and the measurement of CODS. There was a significant difference found in terms of body height, with basketball players being taller than handball players (*p* = 0.032). Professional female handball players achieved better results than professional female basketball players with regard to RA tasks (*p* = 0.01) and CODS (*p* = 0.041). Significant simple correlations between each anthropometric feature (body height, body mass) and values for CODS and RA were observed (*r* = 0.49–0.53). Applying partial correlation allowed for the assessment of actual relationships among CODS, RA, RT, and REAC-INDEX, without a confounding variable. Detaching the anthropometric parameters from the rest of the relationships resulted in maintenance or changes in *r*-values and an increased significance in the relationships between each pair: RA vs. RT, RA vs. REAC-INDEX, and RT vs. REAC-INDEX. The strongest associations were related to RT vs. REAC-INDEX (*r* = 0.97 at detaching body height or body mass, *p* < 0.001) and CODS vs. RA (*r* = 0.66 at detaching body height and *r* = −0.67 at detaching body mass, *p* < 0.001). It is recommended to use partial correlations in subsequent studies, as simple correlations are not reliable and may not reveal the apparent relationships between the variables. In addition, when determining the CODS and RA, it is suggested to take anthropometric and perception variables into account, such as reaction time or REAC-INDEX.

## Introduction

The ability to accelerate, stop quickly, turn or change of direction (COD), and accelerate again is an essential part of the motor skills of a handball and basketball player (Scanlan et al., 2015; Bayraktar, 2017; Šimonek et al., 2017; Conte et al., 2020). This ability is referred to as COD if the movement does not require a response to a stimulus, and it is usually classified as a preplanned and closed skill (Brughelli et al., 2008; Sheppard et al., 2014; Young et al., 2015a). During COD maneuvre, the running phase is followed by a slowdown or stopping phase due to eccentric muscle contraction and the COD. This phase includes adjusting the support (foot contact with the ground in the lateral part of the foot/forefoot) in relation to the center of mass (COM) in order to effectively use the external force to accelerate in the new planned direction of movement (Spiteri et al., 2013; Jones et al., 2017; Dos'Santos et al., 2018). This can be carried out at different speeds depending on the situation on the court. The term “change of direction speed” (CODS) is often used, and it is defined as the ability to COD in the shortest possible time into a predetermined location and space on the field, pitch, or the court (Young et al., 2015a).

In many situations during a game, the players cannot usually plan their movement pattern in advance. This is because their movement constitutes a reaction to an unpredictable single or complex external stimulus (e.g., an opponent, a teammate, ball, etc.). Taking this into consideration, fast and accurate reactions in the form of changes of direction movement performed in response to specific external stimuli are defined as reactive agility (RA) and require a significant involvement of the cognitive-perceptual components of decision-making, such as visual processing, recognition of the space, reaction time, perception, and anticipation (Jones et al., 2009; Spasic et al., 2015; Šimonek et al., 2017). However, CODS remains the physiological and mechanical basis underpinning agility in handball and basketball. Biomechanical studies indicate that preplanned COD movements place less load on the knee compared to a reaction to a stimulus requiring sidestepping, which reduces the risk of lower limb injury (Nimphius et al., 2016; Thomas et al., 2018).

The CODS and RA are the important components of motor performance of a team sports player, and therefore the relationships between them, as well as with other components, should be explored (Pehar et al., 2018). The CODS and RA may be impacted by other factors, such as: anthropometric parameters, training experience, playing level or player position, which should be considered when evaluating these attributes (Sekulic et al., 2014b; Scanlan et al., 2015; Coh et al., 2018, 2019; Pehar et al., 2018; Barrera-Domínguez et al., 2020; Popowczak et al., 2020). Elite players should demonstrate a high level of CODS and RA performance, which allows them to act effectively during a game in planned situations and in response to a sudden external stimulus (e.g., passing the ball, approaching an opponent). At the same time, the question arises whether CODS and RA performance are related? If so, are these dependencies direct or spurious, that is, caused by other variables? There are no answers to these questions in available literature on the subject.

Many studies have shown anthropometric parameters to be related to both CODS and RA (Chaouachi et al., 2009; Young et al., 2015b), due to the importance of body dimensions in the results of motor tests, that is, body height or legs length (Koltai et al., 2021). The explanation is, for example, the lower position of the COM, which determines the efficiency of the COD test, and which is lower in the case of shorter players (Young et al., 2002).

There have been numerous attempts to assess CODS and RA under training conditions; however, few of these are based on the same movement pattern. A uniform pattern in planned and unplanned activities allows one to define not only the physical component but also the perceptual-cognitive component. Based on the study conducted by Spasic et al. (2015) and Morral-Yepes et al. (2020), two movement scenarios for RA and CODS were distinguished, which are specific to basketball and handball players, that is, “stop-and-go” and “SpeedCourt©.” In turn, based on these scenarios, various tests were carried out, differing in the number of changes of direction, the angle of direction change, execution time, and sprint distance between changes of direction (Scanlan et al., 2015; Born et al., 2016; Šimonek et al., 2016; Coh et al., 2018; Popowczak et al., 2020; Peric et al., 2021). The patterns of such actions are most often presented by players during a game, as evidenced by the number of changes of direction per minute performed by players (Luteberget and Spencer, 2017; Svilar et al., 2019; Salazar et al., 2020). Moreover, additional and very helpful indicators for determining RA have been introduced. The first is the index of perceptual and reactive capacity (P&RC index), as a ratio of the participant's performance in the CODS and RA (Spasic et al., 2015). Another is the reactivity index (REAC-INDEX), as the difference between the RA and CODS scores (Fiorilli et al., 2017).

Assessment of the physical abilities in COD and the cognitive motor skills in RA tests is important to understand the ability of players to “read and react” to sport-specific stimuli. Moreover, it is necessary for understanding the physical requirements and capabilities of professional handball and basketball players (Pereira et al., 2018). Nonetheless, at present, there is limited information on COD ability and RA in professional level handball and basketball players, especially regarding differences in the level of these abilities depending on one's sports discipline. Very slight differences in the level of agility (using the Fitro Agility Check test) among young basketball and handball players were observed in the study by Šimonek et al. (2017). In addition, in the aforementioned study, as well as in the study by Silva et al. (2013), there were very small or no differences (*p* > 0.05) in the level of CODS (using the 4 m Shuttle Run test) among the players of these sports. Different results were obtained by Bilge et al. (2020), who concluded that young basketball players achieved shorter times of CODS (*p* < 0.001) than their peers, which was also seen in handball training (as shown by the *T*-test). However, in a study performed by Freitas et al. (2020), it was found that female handball players were faster in the Zig-Zag CODS test (*p* < 0.05) than players of other team sports (for rugby: effect size (ES) = 1.19, for soccer: ES = 1.14). On the other hand, there are no studies that measure CODS and RA in professional basketball and handball players based on tests of specific stop-and-go movement patterns.

Taking into consideration the importance of COD ability and RA in the aforementioned sports, coaches and practitioners have become interested in valid and reliable assessments of these abilities to determine the strengths and weaknesses of their athletes, so that informed decisions can be made regarding the future training proposed for a given player (Thomas et al., 2018). Thus, the main goal of this study was to determine the differences between professional female basketball and handball players in anthropometric features, CODS, and RA task. Moreover, we attempted to determine the relationship among anthropometric variables, CODS, RA, and perceptual parameters. We hypothesized that basketball and handball require athletes to have different performance in terms of CODS, RA, and REAC-INDEX in a task based on a “stop-and-go” movement patterns. As a result, the level of the aforementioned features between professional groups of basketball and handball players will be differentiated.

## Materials and Methods

### Participants

The study group consisted of 31 professional female athletes, including 12 basketball players (mean age: 24.98 ± 3.38; 95%: 22.83 – 27.14) and 19 handball players (mean age: 27.34 ± 4.68; 95%: 25.09–29.6). All the basketball players belonged to the same team, competing in two basketball leagues, that is, the Polish Basketball League (EnergaBasket Liga, 1st League in Poland) and EuroCup Women, in the 2018–19 season. On the other hand, all the handball players belonged to the same team, competing in two handball leagues, that is, the Polish Women's Super League (1st League in Poland) and the Women's EHF Cup, in the 2018-19 season. The study was carried out 1 week after the end of the preseason and was approved by the Research Bioethics Committee of the Faculty Senate of the University School of Physical Education in Wrocław, Poland (reference number: USPE-2013-06-07). The study was conducted in accordance with the ethical principles for medical research involving human subjects contained in the Declaration of Helsinki, developed by the World Medical Association. The study also met the “ethical standards in sport and exercise science research” (Harriss and Atkinson, 2015). All participants were asked to provide written informed consent prior to participation in the study.

### Measures and Procedures

All tests were performed in sports hall facilities, where the athletes participate in league matches and train. Prior to the commencement of physical tests, anthropometric parameters were measured in the morning. All the anthropometric measurements were performed by the same experienced researchers. The height of the participants was measured with a GPM 101 anthropometer (DKSH, Zurich, Switzerland) with a precision of 1 mm. Body mass was measured when the participants were shoeless and wearing minimal clothes using the InBody 230 system (Tanita Corp., Tokyo, Japan).

The physical tests were all performed between 10:00 and 12:00 p.m. Before the measurements, the participants underwent a standardized 15-min warm-up procedure consisting of 5 min of low-intensity running, 5 min of dynamic stretching, and 5 min submaximal running plus COD exercises, multi-jump exercises, and sprints. The participants were then familiarized with the Five-Time Shuttle Run to Gates test.

A Fusion Smart Speed System (Fusion Sport, Coopers Plains, QLD, Australia) was used during the Five-Time Shuttle Run to Gates test (to measure the CODS and RA times). The system is comprised of five gates, each equipped with a photocell with an infrared transmitter and a light reflector, a Smart Jump mat integrated with a photocell and a radio frequency identification reader (RFID) for identification of the participants, as well as computer software (Fusion Smart Speed System application). The layout of the gates, the mat, and the RFID in the Five-Time Shuttle Run to Gates test was adopted on the basis of the previous article (Popowczak et al., 2016). The Fusion Smart Speed System application was used for fixed (preplanned) or random (unplanned) selection of a gate, where the lamp was turned on according to the procedures proposed by Popowczak et al. (2016). The “stop-and-go” scenario of tests as a reaction to a light signal is characterized by reliability levels at an intra-class correlation coefficient (ICC) > 70% (Paul et al., 2016; Morral-Yepes et al., 2020).

The participant had to run the distance from the starting point on the mat to the gate line (placed between the photocells with reflectors, 1 m long) five times and return to the mat. As soon as both feet were in contact with the central part of the mat, the participant received a light signal indicating the gate they should run to. The start to the gates was not delayed. The participant then ran to the line in the gate with a light signal. After crossing it with both feet, the participant returned to the mat. Again, when both feet touched the mat, the participant received another light signal indicating the gate to which they should run. They then repeated the run with a COD four more times. After crossing the last (fifth) gate line, the participant returned to the mat and finished the test. The testing apparatus measured the running time with an accuracy of 0.001 s. The data from the tests was recorded in a personal digital assistant (PDA, HP iPAQ 112).

In the first scenario of the Five-Time Shuttle Run to Gates Test (preplanned), which determines CODS, the participant ran to the gates in an order that was the same for all participants (1-2-3-4-5). The angle of COD was ≅ 180°, while the action on the Mat Jump was performed at an angle of ≅ 135°. The CODS test was repeated twice, and the best result (overall duration) of the run was used in the analysis of the results.

On the other hand, in the second scenario of the Five-Time Shuttle Run to Gates Test (unplanned), determining RA, the run to randomly selected goals was investigated. Their order was different for every participant, but they all covered the same distance. During the RA, participants were instructed not to try to predict which exit gate they would be required to sprint through. The RA test was repeated twice, and the best result (overall duration) of the run (RA) was used in the analysis of the results.

In addition, the REAC-INDEX, which represents the time differences between the RA test result and the measurement of CODS of a similar pattern and for similar distances (Fiorilli et al., 2017), was determined.


$$\begin{array}{l} \text{REAC}-\text{INDEX}\left[\text{s}\right]=\text{RA}\left[\text{s}\right]-\text{CODS }\left[\text{s}\right] \end{array}$$

The average response time (RT) to the first light signal in each RA test was also calculated.

### Statistical Analysis

The Shapiro–Wilk test was used to evaluate the normality of the distribution of the continuous variables. All the variables showed a normal distribution. Descriptive statistics are presented as means, SDs, and 95% CIs.

Unpaired *t*-tests of students were used to evaluate the differences between the two groups of athletes. Cohen's *d* effect size (ES) and respective 95% confidence intervals were also calculated to assess the observed effects (Cohen, 1998). The thresholds of ES were: ≤ 0.2, trivial; 0.2–0.59, small; 0.6–1.19, moderate; 1.2–1.99, large; and ≥2.0, very large (Hopkins et al., 2009).

Pearson's product-moment correlations were used to examine which variables were correlated. The magnitude of the correlation (r) between test measurements was interpreted as: ≤ 0.1–trivial; >0.1–0.3—small; >0.3–0.5—moderate; >0.5–0.7—large; >0.7–0.9—very large; and >0.9–1.0—almost perfect (Hopkins et al., 2009).

Additionally, partial correlations were computed for more insight into the relationships between variables statistically significantly correlating with each other. In this publication, partial correlation was computed for a statistically significant relationship between anthropometric variables and CODS, RA, RT, and REAC-INDEX. Thus, for example, the relationships between both tests (CODS or RA) and body height were determined using partial correlation controlling the effect of body mass; on the other hand, the relationships between both tests (CODS or RA) and body mass were determined using partial correlation controlling the effect of body height. In addition, the relationships between CODS vs. RA, RA vs. RT, RA vs. REAC-INDEX, and RT vs. REAC-INDEX were determined using partial correlation controlling the effect of body height and body mass.

The significance level was set at α = 0.05. Statistical analysis was performed using the application Statistica v.13.0 (StatSoft Polska, Kraków, Poland).

## Results

Descriptive statistics of the parameters for basketball and handball players, as well as *t*-values and *p*-values from the unpaired *t*-test of students, are presented in Table 1. The analysis revealed statistically significant differences between sports in terms of body height, CODS time, and RA time.

**Table 1:** Characteristics of participants by sport disciplines with means, SDs, and 95% CIs.

**Variables**		**Sport**		***t*** **value**	***p***	**Effect size**
		**Basketball**	**Handball**			
Body height [cm]	Mean ± SD	181.67 ± 7.41	175.77 ± 6.93	2.25	**0.032**	0.829
	(95% CI)	(176.96–186.38)	(172.44–179.11)			0.078–1.581
Body mass [kg]	Mean ± SD	74.38 ± 8.54	71.13 ± 8.35	1.05	0.304	0.386
	(95% CI)	(68.95–79.8)	(67.1–75.15)			−0.343–1.115
CODS [s]	Mean ± SD	15.92 ± 0.55	15.31 ± 0.87	2.14	**0.041**	0.798
	(95% CI)	(15.57–16.26)	(14.89–15.73)			0.048–1.547
RA [s]	Mean ± SD	18.37 ± 0.74	17.57 ± 0.87	2.63	**0.014**	0.972
	(95% CI)	(17.9–18.84)	(17.15–17.99)			0.21–1.734
RT [s]	Mean ± SD	0.48 ± 0.15	0.45 ± 0.11	0.54	0.590	0.237
	(95% CI)	(0.38–0.57)	(0.4–0.5)			−0.488–0.962
REAC-INDEX [s]	Mean ± SD	2.45 ± 0.69	2.26 ± 0.54	0.87	0.391	0.316
	(95% CI)	(2.01–2.89)	(1.99–2.52)			−0.411–1.043

*CODS, change of direction speed; RA, reactive agility; RT, reaction time; REAC-INDEX, difference CODS vs. RA, bolded p-value represents significant difference (p < 0.05)*.

Handball players displayed a significantly lower total time in both CODS (*p* = 0.041, ES = 0.789) and RA (*p* = 0.014, ES = 0.972) tests compared to basketball players. However, the height difference was statistically significant. Therefore, it is interesting to analyze the relationship between height and the results of CODS and RA tests. The presence of such a relationship would have a decisive influence on the direction of the result interpretation. In the next step of the study, the correlation coefficients between individual variables were presented (Table 2).

**Table 2:** Matrix of correlation between measured variables.

		**Body mass**	**CODS**	**RA**	**RT**	**REAC-INDEX**
Body height	*r*	0.81	0.49	0.53	0.17	0.12
	*p*	**<0.001**	**0.005**	**0.002**	0.37	0.52
	Body mass	*r*	0.55	0.52	0.09	0.04
		*p*	**0.001**	**0.003**	0.65	0.84
		CODS	*r*	0.76	−0.19	−0.21
			*p*	**<0.001**	0.31	0.25
			RA	*r*	0.48	0.48
				*p*	**0.006**	**0.007**
				RT	*r*	0.97
					*p*	**<0.001**

*CODS, change of direction speed; RA, reactive agility; RT, reaction time; REAC-INDEX, difference CODS vs. RA; r, Pearson r coefficient, bolded p-value represents significant correlation (p < 0.05)*.

Pearson's product-moment correlation coefficient showed large associations between the anthropometric parameters (body height and body mass: *r* = 0.81, *p* < 0.001; body mass and BMI: *r* = 0.71, *p* < 0.001), as well as between CODS and RA (*r* = 0.76, *p* < 0.001), RT and REAC-INDEX (r = 0.97, *p* < 0.001; Table 2). Moderate correlations between both anthropometric parameters and CODS time and RA time were also observed (CODS vs. body height: r = 0.49, *p* = 0.005; CODS vs. body mass: *r* = 0.55, *p* = 0.001; RA vs. body height: *r* = 0.53, *p* = 0.002; RA vs. body mass: *r* = 0.52, *p* = 0.003). Moreover, there was a small correlation between RA and RT on a light signal (*r* = 0.48, *p* = 0.006), as well as between RA and REAC-INDEX (*r* = 0.48, *p* = 0.007).

Based on the results of the simple correlation, strong relationships of anthropometric parameters with the maneuvrability variables of CODS and RA tests were found (Table 2). In order to perform a deeper analysis aimed at determining the importance of each of the two anthropometric parameters in shaping the strength of dependencies between anthropometric, as well as agility and perceptual parameters, a series of partial correlation analyses was performed (Table 3). It was found that close first-order correlations, between one morphological feature and CODS time or RA time, excluding the second morphological feature, are not significant. When controlling the effect of body height, significant moderate-almost perfect correlations were found between CODS and RA (*r* = 0.67; *p* < 0.001), RA and RT (*r* = 0.47; *p* = 0.009), RA and REAC-INDEX (*r* = 0.49; *p* = 0.006), and RT and REAC-INDEX (*r* = 0.97; *p* < 0.001). Controlling the effect of body mass, significant moderate-almost perfect correlations were found between CODS and RA (*r* = 0.66; *p* < 0.001), RA and RT (*r* = 0.47; *p* = 0.004), RA and REAC-INDEX (*r* = 0.49; *p* = 0.002), and RT and REAC-INDEX (*r* = 0.97; *p* < 0.001).

**Table 3:** Partial correlations for the measured variables.

**Relations**	**Correlation between variables**	**Controlling variable**	***r***	***p***
CODS vs. body height or body mass,	CODS vs. body height	Body mass	0.10	0.59
controlling body height or body mass.	CODS vs. body mass	Body height	0.29	0.12
RA vs. body height or body mass,	RA vs. body height	Body mass	0.21	0.27
controlling body height or body mass.	RA vs. body mass	Body height	0.20	0.31
CODS vs. RA,	CODS vs. RA	Body height	0.67	**<0.001**
controlling body height or body mass.	CODS vs. RA	Body mass	0,66	**<0.001**
RA vs. RT,	RA vs. RT	Body height	0.47	**0.009**
controlling body height or body mass.	RA vs. RT	Body mass	0.51	**0.004**
RA vs. REAC-INDEX,	RA vs. REAC-INDEX	Body height	0.49	**0.006**
controlling body height or body mass.	RA vs. REAC-INDEX	Body mass	0.54	**0.002**
RT vs. REAC-INDEX,	RT vs. REAC-INDEX	Body height	0.97	**<0.001**
controlling body height or body mass.	RT vs. REAC-INDEX	Body mass	0.97	**<0.001**
RA vs. RT or REAC-INDEX,	RA vs. RT	REAC-INDEX	0.08	0.67
controlling RT or REAC-INDEX	RA vs. REAC-INDEX	RT	0.06	0.77

*CODS, change of direction speed; RA, reactive agility; RT, reaction time; REAC-INDEX, difference CODS vs. RA; r, Pearson r coefficient, bolded p-value represents significant correlation (p < 0.05)*.

As moderate total correlations were found between RA and RT or REAC-INDEX, the partial correlation was investigated in order to rule out a false relationship. It was found that the analysis of the relationships of each pair of variables does not produce a complete picture of the actual relationships. The analysis of the partial correlations confirms that the study of the relationships of two variables, e.g., RA vs. RT, without considering the related variable REAC-INDEX (*r* = 0.08; *p* = 0.67), produces spurious correlations. This is similar for the relationship RA vs. REAC-INDEX, without considering RT (*r* = 0.06; *p* = 0.77).

## Discussions

The aim of this study was to determine the differences between professional basketball players and handball players in terms of CODS and RA using the “stop-and-go” test scenario. It should be mentioned that this is the first study of professional female basketball and handball teams concerning planned and unplanned changes in running directions using the “stop-and-go” scenario. Moreover, the obtained results cannot be compared to previous studies in which CODS and RA times in team sports were measured.

Based on the obtained results, differences in height (*p* = 0.032, ES = 0.829) were found between professional female basketball players and handball players. Statistically significant differences were observed in terms of CODS (*p* = 0.041, ES = 0.798), as well as RA (*p* = 0.014, ES = 0.972). Handball players obtained better results in tests examining both parameters. Moreover, simple correlation indicated that the taller the player, the longer the execution time of the tests performed to determine CODS and RA. Anthropometric parameters, such as height or leg length, can affect the characteristics of CODS, as shorter individuals take less time to lower their COM (Barrera-Domínguez et al., 2020). Therefore, the involvement of anthropometric parameters in CODS time and RA time were observed. Height specifically affected the results of tests engaging perceptual skills. This observation is of great importance from the point of view of both selection and training itself, and the morphological component seems to be very important for both motor skills.

The results of the analysis clearly demonstrate the correlation between the two morphological variables and CODS. Similarly, morphological variables were correlated with RA. This suggests the need to study the differences in the results of motor ability tests between groups of athletes of various sports disciplines, considering the control of anthropometric parameters (body height and body mass, in our case). The first-order correlations between one morphological feature and CODS or RA, excluding the second morphological feature, are significantly lower than the total correlations. However, a greater role in the formation of a correlation with CODS is played by body mass, or its control consisting of shortening the step and lowering the COM in planned movements (Sattler et al., 2015). In the case of the correlation with RA, the contribution of both anthropometric parameters is similar.

In contrast, Garcia-Gil et al. (2018) observed a non-significant relationship between anthropometric parameters (body height and body mass) and CODS using the *t*-test in professional Spanish female basketball players. The variations in the study findings may concern the movement patterns performed in the respective analyzed tests. In our CODS test, there is a nine-fold COD that lowers the COM, and body height and body mass may have had a significant role in the maneuvring speed and overall test duration. That is why it is so important to select a test for the determination of CODS according to the specific nature of sports disciplines and their dominating movements (maneuvres). Considering the situational and general nature of the Five-Time Shuttle Run to Gates Test for team sports games, future research should continue to investigate the relationship of the test result and various anthropometric parameters in order to determine whether the anthropometric parameters determine the result of CODS during the run more than the type and the specificity of the discipline, as well as the position on the court.

Despite numerous similarities in the performance of actions to COD in a game as previously planned movements, and as a result of a reaction to unpredictable single or complex external stimuli (e.g., an opponent, ball, etc.), athletes of both disciplines showed differences in the time of performing the COD test.

The applied RA test can be classified as a general test measuring the situational ability of athletes of various sports disciplines to COD quickly. This is not a test specific for a given team sport, as it measures the reaction to a light signal and not to the ball or an opponent or a teammate. The differences in the results obtained in RA tests with different reaction stimuli were presented by Scanlan et al. (2016) and Kovacikova and Zemková (2020). Nevertheless, an increase in the level of the ability to COD in response to a light signal may lead to an increase in the level of these abilities in another COD and RA task (Nygaard Falch et al., 2019).

A high correlation coefficient (*r* = 0.76, *p* < 0.001) between RA and CODS was obtained in the present study. Similar results (r from 0.62 and 0.68, *p* ≤ 0.05) were found in the study by Sekulic et al. (2014a) representing various sports, including college-age basketball and handball players. Moderate correlations between CODS and RA (*r* = 0.51 and 0.65, *p* < 0.05) were also observed by Sattler et al. (2015) in college-age athletes (females and males; 21.9 ± 1.9 years of age) representing team sports (football, basketball, volleyball, and handball). On the other hand, weak to moderate correlations (r from 40 to 56, *p* < 0.01) between basketball-specific COD tests and basketball-specific non-planned agility tests were found by Sekulic et al. (2017) in high-level male basketball athletes from Bosnia and Herzegovina (professional/semiprofessional players). Lockie et al. (2014) found weak correlations (*r* = 0.28 and 0.48, *p* < 0.05) or no significant correlations between CODS and RA by examining semiprofessional and amateur male basketball players (22.30 ± 3.97 years of age). However, the large diversity of the information obtained based on research concerning the relationship between CODS and RA and the increasing number of proposed tests require a search for the best correlations between the results obtained in the tests and the results determining COD, accelerations, and deaccelerations during games. Although physical activities (with regard to redirecting total body momentum in a new direction as quickly as possible) constitute a large proportion of the time needed to complete RA and CODS tasks, perceptual decision-making processes may alter the level of correlation between tests (Pehar et al., 2018; Krolo et al., 2020). This should affect the level of correlations between CODS and RA. Information concerning the variables in CODS and RA tests may be of high importance in training and conditioning, since this will allow for specific and targeted development of important qualities that will, consequently, improve specific RA or CODS results.

In the present study, a significant relationship (*r* = 0.97, *p* < 0.001) between RT and REAC-INDEX was found, which may indicate that REAC-INDEX is an important factor determining the reaction time to a stimulus during CODS. The increasing time difference between a CODS test and RA test will be strongly related to the reaction time to the light stimulus, that is, to the perceptual factor. Similar conclusions were reported by Zemková and Hamar (2018), who investigated athletes of different sports (ball hockey and soccer), finding an almost perfect influence (*r* = 0.933, *p* = 0,001) of perception, reaction, and decision-making (measured by the reaction time of the double choice) on agility performance. This almost perfect correlation suggests that improving perceptual skills and, at the same time, decision-making speed may be beneficial for increasing RA in athletes. Moreover, the strong relationship between RT and REAC-INDEX may suggest the need to introduce a new variable determining visual perception skills (as a substitute for response time), which is important for the effectiveness of COD actions. However, this requires further research on the information processing speed of an individual, which is an important component of RA (Lockie et al., 2014). In the present study, the presence of false relationships between RA and RT or REAC-INDEX was also noticed based on partial correlations. This may indicate the need to include reaction time in RA tasks and to analyze REAC-INDEX as the difference between RA and CODS results, as well as the need to study partial correlations instead of total correlations.

The study is limited by the absence of an analysis concerning the COD angle in a run during RA tasks and the strength of the lower limbs as predictors of the characteristics of CODS. Different COD angles could result in a different level of involvement of basic motor components, namely force or speed, when changing the direction of movement. Therefore, it seems important to distinguish those tests in which there were numerous changes of direction based on speed (angle ≅ 0° to ≅ 90°) or force (angle ≅ 135° to ≅ 180°; Bourgeois et al., 2017; Nygaard Falch et al., 2019).

Second, our sample was limited to European clubs; therefore, large cohort studies are required to confirm these results across other regions. A diversity in the results of motor ability parameters in female and male basketball players from different regions of the world was indicated by Milanović et al. (2020) and Stojanović et al. (2018).

In addition, our data were limited to anthropometric parameters and results of CODS and RA tests. Exploring other factors (e.g., playing position of athletes, training experience, playing level), which may influence main findings, should also be included (Scanlan et al., 2015; Young et al., 2015a; Sekulic et al., 2017).

The approach adopted in the present study, although very practical, shows only the time measurements of COD in a run based on the “stop-and-go” scenario. In contrast, further research requires the biomechanical study of this complex ability related to the functioning of the neuromuscular system (Sarvestan et al., 2020). Moreover, these outcomes can be used by basketball players to refine their training and assessment methods in order to optimize RA and COD performance.

### Conclusions

Based on the present study, it was observed that professional female handball players achieve better results than professional female basketball players in COD and RA. It is necessary to perform further research concerning the variables that determine these differences. It is assumed that they would include height and body mass. Moreover, it can be concluded that ordinary (total) correlations constitute false relationships and are not reliable. The relationship among CODS, RA, and basic physical traits treated as an entirety should be explored, while eliminating one of these parameters. This lays out new directions for multidimensional research and analysis. In this paper, it was suggested to use two strongly interdependent predictors (RT vs. REAC-INDEX) in the analysis of RA. Their effect on RA is best assessed using partial correlations. We believe that while the present study is not the final word on the issue, it will extend the knowledge in this field and initiate further research. As a result, coaches and sports scientists should consider these relevant and specific differences when designing specific COD and reactive training programs for professional female handball or basketball players.

## Data Availability Statement

The data analyzed in this study are subject to the following licenses/restrictions: The data are owned by Laboratory for Ball Game Studies, Wroclaw University of Health and Sport Sciences, Poland (certificate PCC-CERT No. PW-06305-19X). Requests to access these datasets should be directed to Marek Popowczak, marek.popowczak@awf.wroc.pl.

## Ethics Statement

The studies involving human participants were reviewed and approved by Research Bioethics Committee of the Faculty Senate of the University School of Physical Education in Wrocław, Poland. The patients/participants provided their written informed consent to participate in this study.

## Author Contributions

MP and JD drafted and revised the manuscript. IC and AR revised the manuscript. All authors contributed to the article and approved the submitted version.

## Funding

All funding pertaining to the realization of this study was received internally by the organization (Wroclaw University of Health and Sport Sciences, Poland) of authors.

## Conflict of Interest

The authors declare that the research was conducted in the absence of any commercial or financial relationships that could be construed as a potential conflict of interest.

## Publisher's Note

All claims expressed in this article are solely those of the authors and do not necessarily represent those of their affiliated organizations, or those of the publisher, the editors and the reviewers. Any product that may be evaluated in this article, or claim that may be made by its manufacturer, is not guaranteed or endorsed by the publisher.
